# Air pollution and lung function among susceptible adult subjects: a panel study

**DOI:** 10.1186/1476-069X-5-11

**Published:** 2006-05-05

**Authors:** Susanna Lagorio, Francesco Forastiere, Riccardo Pistelli, Ivano Iavarone, Paola Michelozzi, Valeria Fano, Achille Marconi, Giovanni Ziemacki, Bart D Ostro

**Affiliations:** 1 National Centre for Epidemiology, Surveillance and Health Promotion, Istituto Superiore di Sanità, Viale Regina Elena 299 00161 Rome, Italy; 2 Department of Epidemiology, Rome E Health Authority, Via di Santa Costanza 53 00198 Rome, Italy; 3 Pneumology Department, Università Cattolica del Sacro Cuore, Via Moscati 31 – 00168 Rome, Italy; 4 Department of Environment and Primary Prevention, Istituto Superiore di Sanità, Viale Regina Elena 299 00161 Rome, Italy; 5 California Office of Environmental Health Hazard Assessment (OEHHA), 1515 Clay St., Oakland, CA 94612, USA

## Abstract

**Background:**

Adverse health effects at relatively low levels of ambient air pollution have consistently been reported in the last years. We conducted a time-series panel study of subjects with chronic obstructive pulmonary disease (COPD), asthma, and ischemic heart disease (IHD) to evaluate whether daily levels of air pollutants have a measurable impact on the lung function of adult subjects with pre-existing lung or heart diseases.

**Methods:**

Twenty-nine patients with COPD, asthma, or IHD underwent repeated lung function tests by supervised spirometry in two one-month surveys. Daily samples of coarse (PM_10–2.5_) and fine (PM_2.5_) particulate matter were collected by means of dichotomous samplers, and the dust was gravimetrically analyzed. The particulate content of selected metals (cadmium, chrome, iron, nickel, lead, platinum, vanadium, and zinc) was determined by atomic absorption spectrometry. Ambient concentrations of nitrogen dioxide (NO_2_), carbon monoxide (CO), ozone (O_3_), and sulphur dioxide (SO_2_) were obtained from the regional air-quality monitoring network. The relationships between concentrations of air pollutants and lung function parameters were analyzed by generalized estimating equations (GEE) for panel data.

**Results:**

Decrements in lung function indices (FVC and/or FEV_1_) associated with increasing concentrations of PM_2.5_, NO_2 _and some metals (especially zinc and iron) were observed in COPD cases. Among the asthmatics, NO_2 _was associated with a decrease in FEV_1_. No association between average ambient concentrations of any air pollutant and lung function was observed among IHD cases.

**Conclusion:**

This study suggests that the short-term negative impact of exposure to air pollutants on respiratory volume and flow is limited to individuals with already impaired respiratory function. The fine fraction of ambient PM seems responsible for the observed effects among COPD cases, with zinc and iron having a potential role via oxidative stress. The respiratory function of the relatively young and mild asthmatics included in this study seems to worsen when ambient levels of NO_2 _increase.

## Background

Throughout the 1990s, many epidemiological studies consistently reported adverse health effects at unexpectedly low levels of ambient air pollution [1]. Identification of susceptible sub-populations and mechanisms of effect involved are two clear research priorities [2,3]. Several chronic clinical conditions are good candidates to define the "frail" population susceptible to the acute effects of PM pollution: chronic obstructive pulmonary disease (COPD) including asthma, ischemic heart diseases (IHD), congestive heart failure, heart rhythm disorders, and diabetes [4].

The mechanisms of lung injury caused by particles among people with COPD have been reviewed [5]. The ability of particulate matter to induce oxidative stress in the airways has been proposed as an important biological mechanism [6]. The oxidative stress mediated by particles may arise from direct generation of reactive oxygen species from the surface of particles or from soluble compounds such as transition metals or organic compounds (poly-aromatic hydrocarbons) [7]. Oxidative stress might up-regulate redox sensitive transcription factors (via nuclear factor kappa B, NF-kB) in airway epithelial cells, thus increasing the synthesis of proinflammatory cytokines and resulting in cell and tissue injury [8].

In healthy and asthmatic volunteers, airborne particles increase bronchial responsiveness, airway resistance, and bronchial tissue mast cell, neutrophil, and lymphocyte counts [9]. A specific role for ultrafine particles and metallic content of PM (especially iron) has been advocated [10,11].

The relationship between daily levels of air pollutants and respiratory function in patients with chronic respiratory diseases has been analyzed in various panel studies, with inconsistent results [12,13]. Most studies concern asthmatic children, while far fewer observations relate to changes in peak expiratory flow rate [14-22] or in spirometric flow and volume [23-26] among adult or elderly asthmatics or COPD patients.

We conducted a time-series panel study of subjects with COPD, asthma, and IHD with the aim of answering the following question: have daily fluctuations of selected air pollutants a measurable impact on the lung function of subjects with pre-existing lung or heart disease?

## Methods

### Participant recruitment

Study subjects were selected among outpatients of the Pneumology and Cardiology Departments of the Catholic University Hospital in Rome (UCSC) included in routine clinical follow-up programs. Eligible for the study were residents of Rome, living in census tracts less than 2 km away from one of the six air monitoring stations considered in this study.

A number of clinical criteria was specified for each nosological category. Eligibility for the COPD panel included age from 50 to 80 years, a ratio of forced expiratory volume in one second to forced vital capacity (FEV_1_/FVC) less than 60%, partial oxygen pressure (SpO_2_) at arterial blood oximetry = 60–70 mmHg, normal values of carboxyhemoglobin (COHb), normal acid-base balance, no concomitant IHD, no need of oxygen therapy or breathing apparatus, no pacemaker, no cardiac arrhythmias, diabetes, Parkinson disease or chronic alcohol abuse, and no use of either psycho-chemical drugs or long acting bronchodilators; occasional use of short-acting bronchodilators was allowed.

Admittance to the asthmatic panel was allowed to subjects aged 18 to 64 years, positive at the bronchial reactivity test by hypertonic saline solution, with disease in the mild intermittent stage [27]. Occasional use of β-adrenergic stimulants was allowed, but assumption of steroids or other asthma-preventive drugs (either before, or during the study periods) was not.

Participation in the IHD panel was restricted to subjects aged 40 to 64 years, with stable angina or previous myocardial infarction (at least 1 year prior to recruitment), no concomitant COPD, no use of calcium channel blockers, no pacemaker, no atrial fibrillation (other arrhythmias admitted), diabetes, Parkinson disease or chronic alcohol abuse, and no use of psycho-chemical drugs.

In relation to smoking habits, participation in the asthmatic panel was restricted to never smokers. Never smokers, however, were almost absent from the clinical series of COPD and IHD outpatients; former smokers were then admitted to the COPD and IHD panels if they had given up smoking at least 1 year before enrollment (sustained quitters).

The study protocol was approved by the Ethical Committee of the UCSC. Twenty-nine patients with COPD (7 men and 4 women), asthma (5 men and 6 women) or IHD (6 men and 1 woman) gave their written informed consent to undergo repeated clinical examinations for two one-month periods, in the spring and winter of 1999.

### Study time period

The time period of interest consisted of 67 days in total, from 24 May to 24 June and from 18 November to 22 December 1999. These periods were chosen based on historical time series analyses of air pollution levels in Rome, due to their high variability in air pollutant concentrations.

### Health monitoring

Study subjects were scheduled to be examined three days apart, at home (COPD and IHD panels) or at the Pneumology Clinic of the UCSC (asthmatic panel). Forced vital capacity (FVC) and forced expiratory volume in 1 second (FEV_1_) were measured by spirometry. Spirometries were supervised, and done with the subject in a sitting position and wearing a nose-clip, following the suggestions of the American Thoracic Society [28]. Spirometries were always done in the afternoon (between 4 pm and 8 pm), at least 6 hours after a possible inhalation of short-acting bronchodilators. A heated Fleish tube n. 3 portable spirometer (Biomedin, Italy) was used in the COPD and IHD panels, and a light bell Stead-Wells spirometer (Biomedin, Italy) in the asthmatic group. Between instruments reproducibility, for both FVC and FEV_1_, was within 30 ml and the calibration procedures were regularly performed [28]. Pulmonary function indices used in the analyses are expressed as the percentage of the predicted values based on the subject- specific sex, age, height and weight [29].

Only amongst asthmatics we determined concentrations of nitric oxide (NO) in exhaled breath, an indicator of bronchial inflammation [30], using the analyzer model 280 (Sievers Instruments, USA). Subjects were breathing NO_x _free air prior to this test. Asthmatics were also asked to fill in a brief daily questionnaire collecting information about the occurrence of asthma attacks and β-2 agonist inhalations.

### Study subjects' characteristics

Table 1 describes the characteristics of subjects at entry and the group-distribution of the outcome variables. As expected on the basis of the eligibility criteria, asthmatics were younger than COPD and IHD patients, and the group average values of FVC and FEV_1 _were sensibly lower among COPD cases compared to both IHD and asthmatic subjects. Overall, the 29 study subjects underwent a total of 449 spirometries. Due to dropouts, a variable number of observations per case was available. The average number of repeated observations was 15 in the COPD panel (ranging from 1 to 32 per subject), 24 in the IHD panel (from 12 to 32 per case), and 9 among the asthmatics (from 6 to 18 per person). All IHD patients were regularly treated with aspirin, statins and nitrates. No COPD or IHD patient made use of bronchodilators (short- or long-acting) during the survey periods, while 7 out of 11 asthmatics reported β-2 stimulant inhalations on one or more of the clinical monitoring days. As to previous smoking habits, all COPD patients were sustained quitters, all asthmatics were never smokers, while in the IHD panel the five male patients were sustained quitters and the single female participant was a never smoker.

**Table 1:** Characteristics of study subjects and group averages of lung function parameters over the survey periods

		**Panel**								
		
		**COPD**			**Asthma**			**IHD**		
	
**Variable **(unit)	**Gender**	**Obs**	**Mean**	**SD**	**Obs**	**Mean**	**SD**	**Obs**	**Mean**	**SD**
**Age **(years)	Male	7	67	11	5	33	15	6	63	11
	Female	4	65	7	6	48	10	1	64	-
	**Total**	**11**	**67**	**9.1**	**11**	**41**	**14**	**7**	**64**	**10**
**BMI **(kg/m^2^)	Male	7	27	6	5	25	4	6	26	3
	Female	4	25	3	6	23	3	1	29	-
	**Total**	**11**	**26**	**5**	**11**	**24**	**3**	**7**	**26**	**3**
**FVC **(% predicted)	Male	108	63	10	38	113	11	139	81	10
	Female	63	61	9	70	116	12	31	80	4
	**Total**	**171**	**63**	**10**	**108**	**115**	**12**	**170**	**81**	**9**
**FEV**_1 _(% predicted)	Male	108	45	10	38	94	13	139	84	10
	Female	63	47	10	70	96	18	31	84	5
	**Total**	**171**	**45**	**10**	**108**	**95**	**16**	**170**	**84**	**10**
**NO in exhaled breath **(ppb)	Male				37	66	40			
	Female				70	42	27			
	**Total**				**107**	**50**	**34**			

### Environmental data

Mean daily temperature (T, Celsius), barometric pressure (BarP, mmHg) and relative humidity (RelHum, %) were available from the Rome weather station (Collegio Romano – Ufficio Centrale di Ecologia Agricola). The Department of Environment of the Latium Region provided us with hourly concentrations of nitrogen dioxide (NO_2_), carbon monoxide (CO), ozone (O_3_), and sulphur dioxide (SO_2_) recorded at the fixed sites for air-quality monitoring in Rome. These sites are equipped with continuous inlet samplers. NO_2 _is determined by chemiluminescence, CO by IR absorption, O_3 _by UV absorption, and SO_2 _by UV fluorescence.

We computed daily city means (24 h values, from 3 pm to 3 pm of the following day) based on data from varying type and number of sites, depending on the pollutant. For NO_2 _and CO, we calculated 24 h values from five fixed sites, three of which are located in densely populated areas in the center of Rome (Magna Grecia, Fermi, Libia) and two representing background areas (Preneste and Villa Ada). For O_3_, concentrations recorded at two background fixed sites (Preneste and Villa Ada) were used. For SO_2_, we used 24 h concentrations recorded at one urban site (Fermi) and one background site (Villa Ada). The NO_2 _and SO_2 _series were complete, while average daily concentrations for one single day were missing for CO and O_3_.

As to particulate matter, we could not use data from the Rome air-quality monitoring network, because PM_2.5 _was not routinely measured. Therefore, for the specific purposes of this survey, 24 h concentrations of PM_10–2.5 _and PM_2.5 _were measured at two selected fixed monitoring sites: Villa Ada and Istituto Superiore di Sanità (ISS). These sites, located about 3.5 km apart, were chosen because, based on historical PM_10 _monitoring data, are considered representative of low and high traffic areas in Rome, respectively. Air samples were collected by means of dichotomous samplers (Graseby Andersen, model SA 241) operating at 16.7 L/min, with an omni-directional aerosol inlet. This sampler has been designated as reference for PM_10 _by US EPA [31]. Sampling was carried out from 3 pm to 3 pm of the following day (in order to match the spirometry time schedule). Sixty-two 24 hour samples were collected, with 5 missing observations at the beginning of the winter survey. The dust on the couple of sequential polytetrafluoroethylene (PTFE) filters (polymethylpentane ringed, 2.0 μm pore size, 37 mm diameter; Gelman, USA) was gravimetrically analyzed to obtain average daily concentrations of PM_2.5 _and PM_10–2.5_. PM_10 _concentrations were calculated by adding the concentrations of the sampled fine and coarse fractions. The averages of PM concentrations measured at the two locations were used in the statistical analyses, as our best estimate of 24 h mean ambient concentrations for the Rome neighborhoods the panel participants lived in.

In the PM_10–2.5 _and PM_2.5 _samples, the content of selected metals (cadmium – Cd, chromium – Cr, iron – Fe, nickel – Ni, lead – Pb, platinum – Pt, vanadium-V, and zinc – Zn) was determined by atomic absorption spectrometry (AAS). The concentrations used in the analysis were calculated as the ratio of the metal amount in each PM sample to the air volume collected during the sampling.

As a side validation study, we measured indoor PM_2.5 _concentrations in a total of five homes of three study subjects per survey. COPD cases were preferentially selected for the side study due to their reduced mobility in comparison with asthmatics and IHD patients. Participation in the side validation study was burdensome to the study subjects: a technician had to come every day to change the sampler filter, and the sampler itself was noisy. Thus, we only succeeded in getting consent to participate from one IHD patient for both surveys, and from four COPD cases, for only one survey each. Participants in the side study were representative of the full study group in terms of housing typology (all lived in apartment buildings), floor [basement, first floor (two homes), second floor, fifth floor], and distance from the ISS or Villa Ada PM_2.5 _monitoring sites (varying from 0.3 to 5 km). Indoor 24 h air samples were collected on 59 days (from 28 May to 24 June and from 22 November to 22 December 1999) by Micro-Environmental Monitors (SKC, model 400) with a single-stage impactor, operating at a sampling flow rate of 10 L/min and equipped with a PM_2.5 _sampling inlet and PTFE filters (polymethylpentane ringed, 2.0 μm pore size, 37 mm diameter; Gelman, USA). Indoor PM_2.5 _mass concentrations were gravimetrically determined. The 24 h average concentrations of PM_2.5_from three homes per survey were used in the reproducibility analysis.

In a previous inter-method reliability study of PM_10 _measurements in outdoor and indoor air samples in Rome, based on two series of 12 parallel 24 h samples, a very good correlation between MEM and dichotomous samplers was observed (regression 1: y = 1.192x-3.275 - R^2 ^= 0.9506; regression 2: y = 0.998x-1.332 - R^2 ^= 0.9866) [32]. According to the European Standard EN 12341 criteria [33], the observed values of the determination coefficients are such that the MEM can be considered equivalent to the dichotomous sampler.

### Statistical analyses

Correlations among outdoor pollutant levels, as well as those between indoor and outdoor PM_2.5 _concentrations, were evaluated by non-parametric tests (Spearman correlation coefficient) applied to variables in the original scale.

Outcome variables in each panel had observations missing, and there was unequal spacing (the interval between observations was not constant). There were a few missing observations in the exposure variables also (1 missing daily mean for both O_3 _and CO concentrations, and 5 missing daily means for PM_2.5 _and PM_10–2.5_). Missing observations, in the exposure or outcome variables, were not replaced with estimates.

The relationships between respiratory function indices and concentrations of air pollutants were analyzed using generalized estimating equations (GEE) for panel data [34]. An autoregressive correlation matrix of lag 1 was assumed, in order to account for possible correlations between repeated measures on the same subject. The statistical package STATA [35] was used for the analyses (XTGEE; the option "force" was specified in order to allow for unequally spaced observations). All the linear models included the within-subject between-period effect, using the dichotomous season variable (spring and winter). For the COPD and IHD panels, terms for daily mean temperature (°C), relative humidity (%) and day of the week (weekday/weekend) were included in the regression models. For the asthmatics panel, temperature and humidity terms were included in the regression models along with β-2 agonist use (yes/no), while the dummy variable weekday/weekend was not, because only 8 out of 108 spirometries were done on Saturday and none on Sunday. We considered the possibility of a non-linear effect of temperature by introducing a temperature-squared term in the regression models; however, since no evidence of model improvement was found in any of the panel-specific analyses, only a linear term was left. The daily variability of pulmonary function was examined with respect to the mean pollutant concentrations of the previous 24 hours and to the cumulative exposures over the previous 48 and 72 hours. Results from the analyses of lung function indices are reported as changes in percentage of predicted values per 10 μg/m^3 ^increase in pollutant concentrations (except for CO where the unit increase is 1 mg/m^3^). In order to assess the relative effects of metals, we report changes in lung function per interquartile range of increasing concentrations.

## Results

### Air pollutant concentrations

The distribution of environmental variable levels (pollutants and weather conditions) during the study period is described in Table 2. Average daily concentrations of both PM_10 _and PM_2.5 _were higher and more variable from day to day in winter than in spring. The two PM fractions did not exceed 123 μg/m^3 ^and 100 μg/m^3^, respectively. The 24 h NO_2 _city means did not show statistically significant differences by season. The daily variability of CO concentrations, on the contrary, was sensibly higher during the winter than in spring survey. As expected, outdoor O_3 _levels were higher and more variable during the spring survey, but never exceeded 100 μg/m^3^. Average 24 h concentrations of SO_2 _were low and showed little variability both in spring and winter. We decided, therefore, *a priori *not to consider SO_2 _further in the analyses. Similarly, we did not include mean daily values of barometric pressure (BarP) in the regression models, due to its negligible daily variability.

**Table 2:** Concentrations of PM, metals from PM_2.5_, gaseous pollutants, and climatic conditions (1999 spring and winter)

**Variable**	**Unit**	**Spring**					**Winter**					**Overall**					
		
		**Obs**	**Mean**	**SD**	**GMean**	**IQR**	**Obs**	**Mean**	**SD**	**GMean**	**IQR**	**Mean**	**SD**	**GMean**	**Min**	**Max**	**IQR**
PM_2.5_*	μg/m^3^	32	18.2	5.0	17.4	8.1	30	36.7	24.1	27.8	40.9	27.2	19.4	21.8	4.5	100	22.7
PM_10–2.5 _*	μg/m^3^	32	18.7	7.4	17.4	9.0	30	12.3	5.4	11.0	8.3	15.6	7.2	14.0	3.4	39.6	7.1
PM_10 _*	μg/m^3^	32	36.9	10.8	35.4	13.1	30	49.0	28.1	40.2	45.3	42.8	21.8	37.6	7.9	123	26.9
**Cd**	ng/m^3^	30	0.29	0.09	0.27	0.14	30	0.63	0.50	0.47	0.47	0.46	0.40	0.36	0.09	2.0	0.31
**Cr**	ng/m^3^	30	0.90	0.47	0.80	0.61	30	2.8	2.0	2.2	2.7	1.9	1.7	1.3	0.2	9.8	1.6
**Fe**	ng/m^3^	30	339	180	303	212	30	227	132	190	208	283	167	240	54.1	893	202
**Ni**	ng/m^3^	30	5.1	4.7	3.6	4.2	30	4.6	8.0	2.7	2.5	4.8	6.5	3.1	1.0	42.3	2.8
**Pb**	ng/m^3^	30	24.4	9.6	22.2	15.6	30	36.8	23.8	27.7	33.5	30.6	19.0	24.8	2.2	99.8	19.9
**Pt**	pg/m^3^	30	5.3	6.8	3.3	3.9	30	4.6	10.3	1.4	2.7	5.0	8.6	2.2	0.05	54.9	3.3
**V**	ng/m^3^	30	2.4	1.6	2.0	2.0	30	1.1	0.52	0.95	0.97	1.8	1.4	1.4	0.47	6.3	1.3
**Zn**	ng/m^3^	30	28.7	13.0	26.4	15.1	30	63.0	38.2	51.4	58.0	45.8	33.1	36.8	13.9	159	32.9
**NO_2 _^†^**	μg/m^3^	32	76.1	13.6	75.0	17.5	35	65.5	13.3	64.0	14.6	70.6	14.4	69.0	27.6	102	20.9
**CO^†^**	mg/m^3^	32	2.1	0.3	2.1	0.50	34	12.3	4.9	11.5	6.1	7.4	6.2	5.0	1.6	28.9	10.5
**O_3 _^‡^**	μg/m^3^	32	70.9	12.5	69.8	16.9	34	16.0	8.1	14.3	10.0	42.6	29.5	30.8	6.6	95.3	58.7
**SO_2 _^‡^**	μg/m^3^	32	4.7	1.8	4.3	2.5	35	7.9	2.2	7.6	3.9	6.4	2.6	5.8	1.2	11.6	4.0
**T**	°C	32	23.7	1.9	23.7	3.3	35	9.5	2.1	9.3	2.7	16.3	7.4	14.5	5.3	27.0	14.3
**RelHum**	%	32	46.5	10.2	45.4	14.0	35	66.7	13.5	65.1	14.0	57.0	15.7	54.8	29.0	87.0	24.0
**BarP**	mm Hg	32	762	2.6	762	3.4	35	762	7.3	762	12.1	762	5.5	762	748	773	6.6
**PM_2.5 _Indoor^§^**	μg/m^3^	28	24.7	7.8	23.7	9.0	31	27.0	12.0	24.4	18.8	25.9	10.2	24.1	10.2	56.5	14.4

*Averages of 24 h concentrations measured at 2 locations.

^†^Averages of 24 h concentrations recorded at 5 fixed sites.

^‡^Averages of 24 h concentrations recorded at 2 fixed sites.

^§^Averages of 24 h concentrations measured at 3 study participants' homes per season.

In terms of relative concentrations, six out of 8 metals examined were more represented in the fine than in the coarse fraction of outdoor PM; therefore only metal concentrations from the PM_2.5 _fraction are reported in Table 2. Fe, Zn and Pb were present in sizeable concentrations in outdoor PM_2.5 _samples, whereas Pt, Cd, V, Cr and Ni were only present in traces.

Correlations among ambient variables are reported in Table 3. Outdoor concentrations of fine (PM_2.5_) and coarse (PM_10–2.5_) particulate matter were weakly correlated. Daily mean levels of PM_2.5 _were directly correlated with barometric pressure, CO and NO_2 _concentrations, inversely correlated with O_3 _and temperature, and unrelated to relative humidity. Average daily concentrations of PM_10–2.5 _were positively correlated with NO_2_, temperature, and to a lesser extent with O_3_, inversely correlated with relative humidity, and not correlated with CO. NO_2 _concentrations were neither correlated with CO, nor with O_3_. Daily mean levels of CO and O_3 _showed a strong negative correlation.

**Table 3:** Correlations among pollutants and weather variables

	**PM_2.5_**	**PM_10–25_**	**PM_10_**	**NO_2_**	**O_3_**	**CO**	**SO_2_**	**T**	**RelHum**	**BarP**
**PM_2.5_**	1									
**PM_10–25_**	0.34	1								
**PM_10_**	0.93	0.61	1							
**NO_2_**	0.43	0.51	0.45	1						
**O_3_**	-0.51	0.31	-0.36	0.17	1					
**CO**	0.67	-0.09	0.55	0.05	-0.87	1				
**SO_2_**	0.34	-0.16	0.21	0.01	-0.61	0.65	1			
**T**	-0.31	0.56	-0.10	0.36	0.79	-0.74	-0.56	1		
**RelHum**	0.17	-0.37	0.06	-0.36	-0.62	0.54	0.43	-0.48	1	
**BarP**	0.65	0.24	0.56	0.47	-0.15	0.28	0.18	-0.15	-0.17	1

Correlations between ambient concentrations of PM_2.5 _and each metal and between metals were also examined (data not shown). PM_2.5 _daily means were highly correlated with Zn, Cd and Pb levels (ρ = 0.778, 0.714 and 0.694 respectively), moderately correlated with Cr, Pt, Ni and Fe (ρ = 0.565, 0.491, 0.475 and 0.463 respectively), and not correlated with vanadium concentrations (ρ = 0.151). Fe concentrations were scarcely correlated with both Zn and Pb (ρ = 0.318 and 0.328), and moderately correlated with Pt (ρ = 0.564). Pt was scarcely correlated with Zn and Pb (ρ = 0.299 and 0.421). Zn and Pb concentrations were moderately correlated (ρ = 0.663).

**Figure 1:**
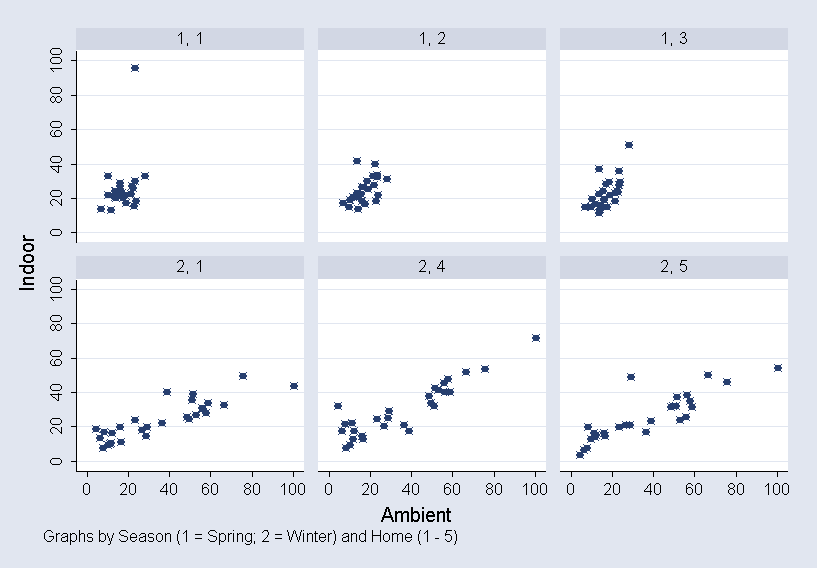
Scatter plot of ambient* PM_2.5 _and indoor** concentrations (μg/m^3^), by season^† ^and home^‡ ^*Averages of PM_2.5 _24 h concentrations (μg/m^3^) at two sites (Villa Ada and ISS). **PM_2.5 _24 h indoor concentrations (μg/m^3^) in each home participating in the side validation study. ^†^Spring = 1 and Winter = 2. ^‡^1 to 5.

Daily indoor concentrations of PM_2.5 _(averages of 24 h samples collected at the homes of three subjects per survey) were highly correlated with average ambient PM_2.5 _(ρ = 0.81, p < 0.01). A reverse PM_2.5 _indoor/outdoor ratio was evident in the two seasons. Concordance was higher during the winter survey (ρ = 0.91, p < 0.01) than in spring (ρ = 0.59, p = 0.01), apparently due to a greater daily variability of ambient PM_2.5 _concentrations in winter than in spring, and not to home-specific characteristics (Figure 1).

### Lung function

We observed a negative association between ambient PM_2.5 _and PM_10 _and respiratory function (FVC and FEV_1_) in the COPD panel (Table 4). The effect on FVC was evident both at a short lag (24 h) and in relation to cumulative exposures over the previous 24 and 48 hours. The effect on FEV_1 _appeared only when 72 hours of exposure were accumulated. A FEV_1 _reduction was also seen with increasing NO_2 _concentrations during the previous 24 and 48 hours. In the asthmatic panel, we observed decreasing values of FEV_1 _related to cumulative exposure to NO_2 _concentrations during the preceding 24, 48, and 72 hours (Table 4). No association between respiratory function indices and average concentrations of any of the pollutants (neither at various lags, nor as cumulative exposure over 48 or 72 h) was observed among IHD patients (Table 4).

**Table 4:** Variations in respiratory function per unit increase of air pollutant concentrations in the three panels

			**PM**_2.5_			**PM**_10–2.5_			**PM**_10_			**NO**_2_			**CO**			**O**_3_		
**Pollutant increase**			**10 μg/m**^3^			**10 μg/m**^3^			**10 μg/m**^3^			**10 μg/m**^3^			**1 mg/m**^3^			**10 μg/m**^3^		
**Panel**	**Outcome**	**Time**	β *	**SE (β)**	**p**	**β **	**SE (β)**	**p**	**β **	**SE (β)**	**p**	**β **	**SE (β)**	**p**	**β **	**SE (β)**	**p**	**β**	**SE (β)**	**p**
**COPD**		**24 h**^†^	-0.80	0.36	0.027	-1.32	1.06	0.210	-0.66	0.30	0.027	-0.72	0.49	0.139	-0.14	0.15	0.353	0.01	0.57	0.983
	**FVC (%)**	**48 h**^‡^	-0.89	0.41	0.031	-1.46	1.31	0.265	-0.75	0.35	0.032	-0.43	0.58	0.463	-0.13	0.18	0.497	0.18	0.65	0.783
		**72 h**^§^	-1.10	0.55	0.044	-1.38	1.53	0.367	-0.94	0.47	0.045	-0.10	0.70	0.886	0.15	0.23	0.508	-0.03	0.86	0.977
		**24 h**^†^	-0.47	0.33	0.149	-0.59	0.95	0.532	-0.37	0.27	0.167	-1.16	0.41	0.005	-0.05	0.13	0.725	-0.20	0.50	0.687
	**FEV**_1_**(%)**	**48 h**^‡^	-0.69	0.37	0.061	-1.01	1.19	0.396	-0.58	0.31	0.067	-1.38	0.49	0.005	-0.12	0.16	0.467	0.31	0.56	0.578
		**72 h**^§^	-1.06	0.50	0.032	-0.90	1.42	0.524	-0.87	0.43	0.040	-0.94	0.60	0.117	-0.03	0.20	0.900	0.68	0.74	0.363
**Asthma**		**24 h**^†^	-0.14	0.29	0.617	-0.17	0.75	0.822	-0.12	0.24	0.621	-0.53	0.31	0.081	0.02	0.12	0.842	-0.33	0.41	0.416
	**FVC (%)**	**48 h**^‡^	-0.07	0.33	0.825	-0.36	0.91	0.695	-0.09	0.29	0.750	-0.59	0.32	0.065	-0.001	0.13	0.995	0.02	0.42	0.957
		**72 h**^§^	-0.06	0.39	0.886	-0.24	1.07	0.824	-0.08	0.36	0.836	-0.54	0.37	0.149	-0.06	0.16	0.700	0.14	0.50	0.782
		**24 h**^†^	-0.30	0.34	0.372	-0.67	0.89	0.448	-0.28	0.28	0.317	-1.10	0.35	0.002	-0.05	0.14	0.704	-0.41	0.50	0.421
	**FEV**_1_**(%)**	**48 h**^‡^	-0.36	0.39	0.347	-1.19	1.07	0.265	-0.40	0.34	0.239	-1.28	0.37	0.001	-0.16	0.15	0.292	-0.01	0.51	0.983
		**72 h**^§^	-0.40	0.46	0.384	-0.51	1.26	0.689	-0.40	0.43	0.351	-1.17	0.44	0.007	-0.28	0.18	0.126	0.46	0.60	0.449
**IHD**		**24 h**^†^	0.37	0.26	0.164	0.37	0.73	0.617	0.28	0.22	0.198	0.15	0.29	0.612	0.176	0.101	0.081	0.58	0.33	0.077
	**FVC (%)**	**48 h**^‡^	0.51	0.29	0.082	-0.25	0.88	0.779	0.35	0.25	0.160	-0.06	0.37	0.868	0.132	0.120	0.271	0.36	0.42	0.392
		**72 h**^§^	0.73	0.40	0.069	0.67	1.04	0.519	0.59	0.34	0.079	0.36	0.47	0.440	0.132	0.165	0.425	0.08	0.60	0.898
		**24 h**^†^	0.43	0.33	0.192	0.25	0.86	0.770	0.32	0.27	0.240	-0.24	0.36	0.513	0.204	0.120	0.088	0.57	0.39	0.144
	**FEV**_1_**(%)**	**48 h**^‡^	0.55	0.34	0.106	-0.23	1.02	0.818	0.38	0.29	0.193	0.00	0.44	0.994	0.114	0.142	0.420	0.53	0.50	0.288
		**72 h**^§^	0.66	0.47	0.157	0.18	1.19	0.877	0.49	0.39	0.214	0.21	0.56	0.710	0.159	0.194	0.412	0.65	0.70	0.354

*Regression coefficient from GEE models for panel data controlling for repeated individual observations, temperature, relative humidity, and day of the week (COPD and IHD panels) or β-2 stimulant inhalation (asthma panel), representing the change in lung function index (% of predicted) per unit increase of pollutant concentration.

^†^Concentration during the previous 24 hours.

^‡^Average concentration over the previous 48 hours.

^§^Average concentration over the previous 72 h.

**Figure 2:**
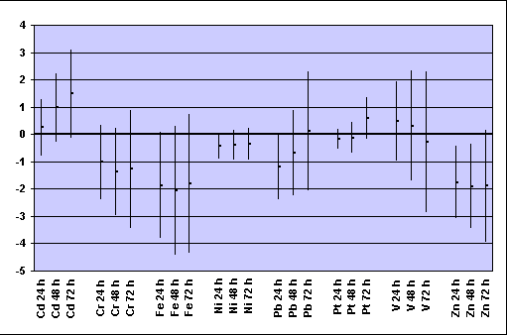
COPD panel: changes in FVC per interquartile increase of selected metals from PM_2.5 _samples during the previous 24, 48 or 72 hours* *Regression coefficients β (dots) and 95% confidence intervals (bars) from GEE models for panel data controlling for repeated individual observations, temperature, relative humidity, and day of the week, representing changes in FVC (% of predicted) per interquartile range increase of metal concentration (see Table 2 for the IQR values).

**Figure 3:**
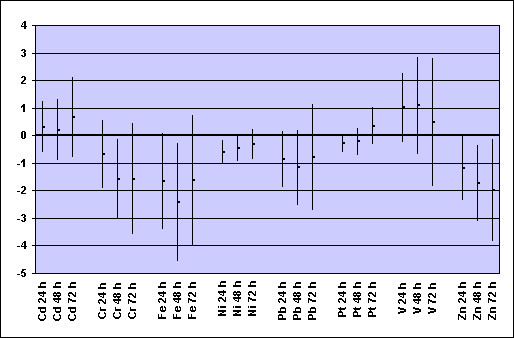
COPD panel: changes in FEV_1 _per interquartile increase of selected metals from PM_2.5 _samples during the previous 24, 48 or 72 hours* *Regression coefficients β (dots) and 95% confidence intervals (bars) from GEE models for panel data controlling for repeated individual observations, temperature, relative humidity, and day of the week, representing changes in FEV_1 _(% of predicted) per interquartile range increase of metal concentration (see Table 2 for the IQR values).

In the COPD panel, Zn concentrations were associated with FVC and FEV_1 _decrements at single 24 h lag and at cumulative 48 h and 72 h lags (Figures 2 and 3). The associations were similar in size but less consistent for Fe and Ni. No statistically significant negative association between concentrations of metals and lung function indices was observed in the asthmatic and IHD panels (data not shown).

In the group of asthmatics, a total of 107 measurements of NO concentrations in exhaled breath were performed. Overall, there was no clear association of the various pollutants with this biological marker of inflammation.

## Discussion

This study suggests an effect of fine particles on lung function of COPD patients. The metallic content of PM_2.5 _seems to be of importance, given the observed negative effects of Zn, Fe and Ni concentrations on lung function indices. NO_2 _also was associated with FEV_1 _decrements among COPD cases. On the other hand, no effects of PM_2.5 _were found among asthmatics, whose respiratory function seemed to be negatively influenced by ambient NO_2 _concentrations. No pollutant-related lung function changes were observed among IHD subjects, with no pre-existing lung impairment.

We acknowledge several limitations of the study. Lack of personal exposure measurements is an important shortcoming. Lifestyles and housing conditions among our study subjects were not homogenous, and we cannot expect outdoor PM_2.5 _concentrations in our study to be a perfect indicator of personal exposure variability, as it was reported among elderly subjects residing in a retirement facility [36,37]. However, the good correlation we observed between day-to-day variations in average outdoor and indoor PM_2.5 _should mitigate the possibility that findings among COPD patients are entirely due to errors in estimating personal exposure variability. Moreover, despite the high number of repeated observations per subject, the reduced number of patients in each diagnostic category gives a low power to the study, thus we cannot exclude the possibility that small effects were not detected because of the reduced sample size. Last, the present study shares with other studies of air pollution related health effects the drawback of multiple statistical testing.

There are however strengths of the study that are worth underlining. They include: (i) supervised lung function tests; (ii) many repeated observations per patient, which allowed accounting for within-subject variability; (iii) a variety of measured urban pollutants, including fine particles, coarse particles and transition metals. Furthermore, in the present study no association between respiratory function indices and average ambient concentrations of any pollutants was observed among IHD cases, whereas effects of the fine fraction of ambient particulate on lung function among COPD subjects were detected, which had a strong *a priori *hypothesis. In the light of such results, it seem less likely that the associations observed among COPD and asthmatic patients are chance findings due to the great number of relationships examined.

After considering validity issues, it is worth noting the results of other published works. To our knowledge, only two studies have evaluated lung function by supervised spirometry in relation to daily variation in air pollution among adults with COPD. In the panel study of Pope and Kanner [25], based on 2 repeated observations of 251 smokers with mild to moderate COPD, a 10 μg/m^3 ^increase in PM_10 _was associated with an average decrease in FEV_1 _equal to approximately 0.2%. Brauer et al. [26], in their panel study of 16 COPD cases with moderate airway obstruction (FEV_1 _at baseline ≥ 0.75 l) observed a non-significant 1.1% decrease of FEV_1 _for 10 μg/m^3 ^increase in ambient PM_2.5_. In four other studies [19-22], possible pollution-related effects on pulmonary function in COPD cases were examined by unsupervised measurements of peak expiratory flow (PEF). To our knowledge, our study is the first that was able to document a specific role of fine particles on lung function of COPD patients.

PM-related exacerbation of chronic obstructive lung disease may be sustained by multiple direct and indirect mechanisms [5,38,39]. There is empirical evidence and experimental support for direct damages to the respiratory mucosa (increased permeability and reduced mucociliar activity), for oxidative damage, and for secondary toxic effects mediated by pro-inflammatory cytokines. Relative to healthy subjects, patients with moderate-to-severe airway obstruction receive an increased dose from ultra fine particle exposure [40]. Transition metals [7,8], as well as ultra fine particles [6], may induce oxidative stress and inflammatory response. We are not aware of previous panel studies of adult COPD cases examining pulmonary function changes in relation to the metallic content of airborne PM. The exploratory analyses presented in this paper suggest that metals from inhaled particulate have a biological effect on pulmonary function. The results on Zn and Fe are of specific interest. Both metals present in the fine fraction are likely to be traffic related as they originate from engine oils, brake, engine, exhaust systems and tire wear [41]. They have a high water solubility which has been directly related to oxidative damage [42].

Results from the asthmatic panel suggest a negative influence of NO_2 _on FEV_1_, but no effect of fine particles. Our finding that NO_2 _is related to lung function decrements both among COPD and asthma patients is of interest. It is difficult to believe that NO_2 _per se is responsible of the observed effects, given its low intrinsic toxicity. It has been suggested, however, that NO_2 _may be considered a very good marker of the combustion mixture from traffic sources, in particular of ultra fine particles [43].

No effect of O_3 _concentrations on the respiratory function of asthmatics was detected, even when the analysis was restricted to the spring survey. The latter finding could be explained by the relatively good clinical conditions of the asthmatics included in the study (all in the mild intermittent stage), the relatively low levels of O_3 _recorded during the study period, or simply lack of power. Although many panel studies of adult asthmatics have reported associations between asthma symptoms and both PM and O_3 _[13,14], inconsistent results were observed with regard to lung function. Moseholm and coworkers [16] found that increased levels of SO_2 _and NO_2 _corresponded synergistically to decreased peak flow at levels above 40 μg/m^3^. In the asthmatic panel studied by Taggart et al. [24], changes in bronchial hyper-responsiveness were significantly correlated with 24 h mean concentrations of SO_2_, NO_2 _and black smoke, none of the criteria air pollutants seemed to affect FEV_1, _while previous-day NO_2 _levels were associated with FVC decrements. Ambient PM_10 _concentrations negatively affected PEF readings among the asthmatics followed by Peters et al. [17], with especially strong effects due to the number of ultra fine particles. Higgins et al. [19] observed PEF decrements associated with SO_2 _and O_3 _among the methacolin-reactors in their panel, but no independent effect of ambient NO_2 _levels. On the other hand, among the asthmatics followed by Hiltermann et al. [18], ambient concentrations of O_3_, PM_10_, black smoke and NO_2 _were found to be associated with increased symptom reports, but not with decreased PEF readings.

## Conclusion

In conclusion our study, despite its limitations, suggests that the short-term negative impact of exposure to relatively low concentrations of air pollutants on lung function is limited to individuals with already impaired respiratory health. The fine fraction of ambient PM seems responsible for the observed effects among COPD cases, with zinc and iron having a potential role. These hints require confirmation from larger and more focused panel studies, using appropriate methods to overcome the problem of multiple comparisons [44].

## Abbreviations

AAS = Atomic absorption spectrometry

BarP = Barometric pressure

BMI = Body mass index [weight (kg) / height^2^(m)]

°C = Celsius degrees

Cd = Cadmium

CO = Carbon monoxide

COHb = Carboxyhemoglobin

COPD = Chronic obstructive pulmonary disease

Cr = Chrome

Fe = Iron

FEV_1 _= Forced expiratory volume in 1 second (% of predicted)

FVC = Forced vital capacity (% of predicted)

GEE = Generalized estimating equations

GMean = Geometric mean

HbO_2 _= Arterial blood oxygen saturation

IHD = Ischemic heart disease

IQR = Interquartile range

IR = Infrared

Mean = Arithmetic mean

MEM = Micro Environmental Monitor

Max = Maximum

Min = Minimum

Mm Hg = Millimeters of mercury

μg/m^3 ^= Micrograms per cubic meterng/m^3 ^= Nanograms per cubic meter

Ni = Nickel

NO = Nitric oxide

NO_2 _= Nitrogen dioxide

O_3 _= Ozone

Obs = Number of observations

PEF = Peak expiratory flow

Pb = Lead

PM = Particulate matter

PM_10 _= Particulate matter with aerodynamic diameter less than 10 μm

PM_10–2.5 _= Particulate matter with aerodynamic diameter between 10 and 2.5 μm

PM_2.5 _= Particulate matter with aerodynamic diameter less than 2.5 μm

Pt = Platinum

PTFE = Polytetrafluoroethylene

RelHum = Relative humidity

SD = Standard deviation

SO_2 _= Sulphur dioxide

SpO_2 _= Partial oxygen pressure

T = Temperature

UCSC = Catholic University Hospital

UV = Ultraviolet

V = Vanadium

Zn = Zinc

## Competing interests

The author(s) declare that they have no competing interests.

## Authors' contributions

SL conceived the study, participated in its design and in data analysis, coordinated its realization, made substantial contribution to the interpretation of results, and drafted the manuscript. FF conceived the study, participated in its design, made substantial contribution to the interpretation of results and participated in drafting the manuscript. RP participated in the design of the study, selected the study subjects and coordinated the clinical monitoring. II participated in data acquisition and data management, and carried out the statistical analysis. PM participated in the design and in the coordination of the data collection. VF participated in the data management and statistical analysis. AM and GZ coordinated the monitoring of atmospheric pollutants and the analysis of environmental samples. BDO participated in design of the study and made substantial contribution in the interpretation of results.
